# Editorial: Novel Mechanisms of Epileptogenesis and Its Inspired Pharmaceutical Treatments for Epilepsy

**DOI:** 10.3389/fneur.2022.942365

**Published:** 2022-06-28

**Authors:** Antonella Riva, Pasquale Striano

**Affiliations:** ^1^Pediatric Neurology and Muscular Diseases Unit, IRCCS Istituto Giannina Gaslini, Genoa, Italy; ^2^Department of Neurosciences, Rehabilitation, Ophthalmology, Genetics, Maternal and Child Health, University of Genoa, Genoa, Italy

**Keywords:** epilepsy, pathogenesis, pharmacotherapy, precision medicine, treatments

Epilepsy is a common chronic neurological disorder affecting approximately 0.5–1% of the population worldwide (~50,000,000 people) and it accounts for a variety of neurological disorders characterized by recurrent seizures. More than half of all epilepsies have some genetic basis and single gene defects in ion channels or neurotransmitter receptors are associated with inherited forms of epilepsy (1). In the last decades, epileptogenic mutations have been identified in several ion channel genes, leading to the concept that several epilepsies can be considered channelopathies (2, 3). Functional studies have in some cases provided significant advances in the understanding of the molecular and cellular dysfunctions caused by mutations. However, the relationships between molecular deficits and clinical phenotypes are still unclear. Sun et al. showed that decreased activity of α7 nicotinic acetylcholine receptors (nAChRs) increases the excitability of CA1 pyramidal neurons and reduces the onset time of epilepsy in pilocarpine-induced mouse models. Moreover, the expression of α7 nAChRs is downregulated in human epileptogenic tissues. Overall, their findings confirm that α7 nAChR is an essential regulator of seizure susceptibility. However, the etiology of epilepsy is extremely complex and heterogeneous and both genetic and acquired factors can be responsible for this condition; the cellular mechanisms underlying the epileptogenicity depend on the integrity of the blood-brain barrier, circuit abnormalities, or cellular and molecular defects, leading to epileptogenesis. Nevertheless, there is still a growing need for the identification of accurate biomarkers of epileptogenesis that enable the prediction of epilepsy following a brain insult. Recent technical progress may offer the opportunity for further investigating cortical areas and brain networks involved in cerebral functions and in epileptic discharges. Chen et al. present a comprehensive overview of recent innovations in the role of neuroimaging and EEG in identifying reliable biomarkers of epileptogenesis, whereas Shen et al. provide a review of the mechanisms of secondary epileptogenesis in molecular, cellular, and circuity levels. Zhang et al., instead, used magnetoencephalography (MEG) to evaluate whether the neuromagnetic signals of the brain neurons correlated with the response to therapy in drug-naïve patients and showed that a local frontal epileptic network at 80–250 Hz may increase the risk of drug-resistance in childhood absence epilepsy.

Despite a tremendous increase in the opportunities for non-invasive research on the human brain animal models of epilepsy still allow the investigation of the mechanisms of epileptogenesis and are also useful to study the consequences and co-morbidities of epilepsy and to develop effective treatments. Wang et al. focus their review on the brain-derived neurotrophic factor (BDNF), a member of the neurotrophic factor family with an important role in the survival, growth, and differentiation of neurons, and discuss the possibility of BDNF as an underlying target for the treatment of epilepsy, whereas Han et al. highlight the potential role of vascular endothelial growth factor (VEGF) as a critical neurovascular target in modulating epileptogenesis in the animal immature brain after lithium-pilocarpine-induced status epilepticus (SE). Moreover, symptomatic SE is one of the highest risk factors of epileptogenesis as shown by the study by Tong et al. who compared the effect of different drugs in the pilocarpine model of acquired epilepsy.

In humans, SE is a medical emergency associated with acute severe systemic damage and high mortality as confirmed by Li et al. describing a rare nonconvulsive status epilepticus following surgical resection of a pituitary tumor and not taking regular hormone replacement therapy. Nevertheless, the challenge of finding new, more efficacious, and better-tolerated drugs is ongoing. The pipeline for the development of new ASMs with novel mechanisms of action is narrowing with only a few interesting compounds on the immediate horizon. Recent studies prompted the identification of neuroinflammation as a potential target for the treatment of epilepsy, particularly drug-resistant epilepsy, and refractory status epilepticus. In Costagliola et al., a systematic review of the clinical experience with anti-cytokine agents and agents targeting lymphocytes is provided offering promising main therapeutic perspectives in this field. Finally, there is also increased interest in the use of possible alternative treatments. Zhong et al. tested the effect of crocin, the main component of Crocus sativus L., in pilocarpine-induced epileptic mice suggesting a potential anti-epileptic property for this natural compound.

In brief, this issue of Frontiers may serve as a helpful guide for epileptologists, including those beginning their careers and honing their skills, as well as for medical students and residents who want to learn more about the pathophysiology, epidemiology and burden, comorbidities, treatment, and research for the management of epilepsy.

## Author Contributions

AR: writing of the manuscript. PS: writing of the manuscript and critical revision of the manuscript. Both authors contributed to the article and approved the submitted version.

## Conflict of Interest

AR has received honoraria from Kolfarma s.r.l, Proveca Pharma Ltd, and PTC Therapeutics. PS has served on a scientific advisory board for the Italian Agency of the Drug (AIFA), received honoraria from GW pharma, Kolfarmas.r.l., Proveca Pharma Ltd, and Eisai Inc., and received research support from the Italian Ministry of Health and Fondazione San Paolo.

## Publisher's Note

All claims expressed in this article are solely those of the authors and do not necessarily represent those of their affiliated organizations, or those of the publisher, the editors and the reviewers. Any product that may be evaluated in this article, or claim that may be made by its manufacturer, is not guaranteed or endorsed by the publisher.
